# Production of Modified Composite Nanofiber Yarns with Functional Particles

**DOI:** 10.1021/acsomega.2c06468

**Published:** 2022-12-22

**Authors:** Josef Skrivanek, Radek Jirkovec, Ondrej Batka, Pavel Holec, Tomas Kalous, Petr Zabka, Martin Bilek, Pavel Pokorny

**Affiliations:** † Department of Textile Machine Design, Faculty of Mechanical Engineering, Technical University of Liberec, Liberec 460 01, Czech Republic; ‡ Department of Nonwovens and Nanofibrous Materials, Faculty of Textile Engineering, Technical University of Liberec, Liberec 460 01, Czech Republic

## Abstract

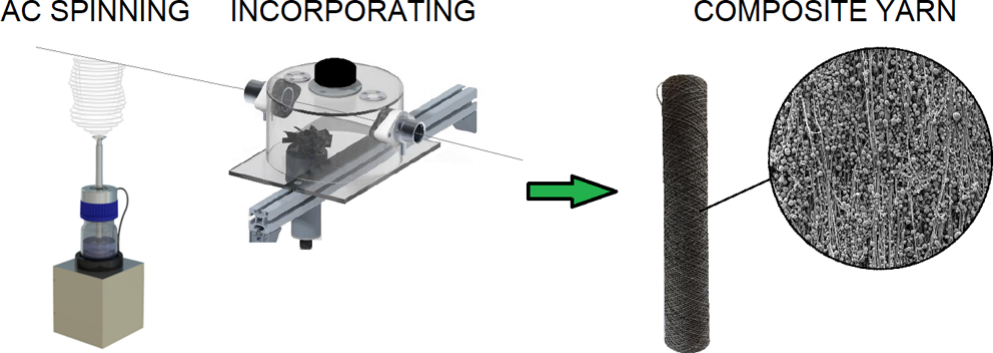

The study focused on the production of modified composite nanofiber yarns with fine functional particles. A device that incorporates fine functional particles into a nanofiber yarn wrapper was specially developed, which ensures the continuous production of modified yarn. It was demonstrated during the study that the specially designed equipment could be used effectively for incorporating fine functional particles into the nanofiber packaging, thus creating a unique yarn with high application potential. The use of particles with dimensions of just tens of micrometers results in the uneven flow of particles inside the chamber and the uneven supply of particles to the composite yarn. The study also determined that the number of particles incorporated into the composite yarn is affected by the particle concentration and the variation of the vortex velocity ratios in the chamber. During testing, it was also found that the nanofiber sheet of the composite yarn improves the mechanical properties of the produced yarn. In addition, the study included the semi-industrial production of a composite filter candle, which can be used for the treatment of fluids, especially air and water.

## 1 Introduction

Nanofibers are defined as ultrafine fibers with a diameter of less than 1000 μm. Due to their unique properties, including their high specific surface area and small fiber diameters and pore sizes, they are widely used for filtration or tissue engineering purposes. However, due to their limited mechanical properties, nanofibers are rarely used alone; rather, they are used in the form of composites in most applications,^1,2^ an example of which comprises the core-sheath composite yarn. This composite yarn consists of two parts, namely, the carrier yarn, that is, the core, and a nanofiber layer, the so-called sheath.^3^ In general, nanofiber layers can be produced via a number of approaches. However, the electrospinning technique, either direct current (DC) spinning^4,5^ or alternating current (AC) spinning,^6,7^ is employed to create the composite yarn. With the help of the supporting core, the composite yarn provides the mechanical parameters required for subsequent technological processing and application. Polyacrylonitrile,^8^ polyvinylidene fluoride,^9^ polyurethane,^9^ and polyvinyl butyral^3^ can be used to create the nanofiber packaging.

The formation of composite DC spinning yarns has been successfully tested via a number of previous experimental studies.^10^ Nevertheless, the main drawback of the DC technique concerns the requirement for a charged/grounded counter electrode.^11,12^ Thus, AC spinning, which does not require a counter electrode, appears more suitable for the fabrication of composite yarns. No counter electrode is necessary since the AC spinning technique relies on constant high-voltage polarity changes that result in the creation of a so-called nanofiber siding which is charged by both positive and negative charges due to changes in polarity. The charges are subsequently compensated for, thus rendering the siding electrically neutral. The nanofiber siding is then carried away from the electrode by the ionic wind.^13,14^ The approach to the creation of composite yarns using the AC spinning approach has been set out in several of our previous publications, in which we described the optimization of the composite yarn production process.^3,6^ Moreover, we have proven that such yarns with a nanofiber layer can easily be processed so as to create other textile structures such as fabrics and knitwear.

Composite yarns are used primarily in the production of filtration products as well as in the health sector^15,16^ and the textile industry for the production of clothing.^17,18^ However, they can be further modified so as to enhance their application potential,^19^ particularly via their combination with other high-surface-area components such as activated carbon (so as to increase their filtration properties).^20,21^ For filter applications, it is possible to use, for example, the widely used polyamide^22,23^ or polyvinyl butyral.^24^ Their potential application in the health sector includes the creation of nanofibrous surgical suture sheaths enriched with antibiotic particles aimed at preventing the spread of microorganisms due to, for example, anastomotic leakage.^25,26^ Yarns can be modified via either the grafting of specific molecules onto the fibers or the addition of functional particles^27^ directly to the spinning solution, the main disadvantage of which is that the particles may not be spun together with the polymer solution and that the spun particles become encapsulated within the polymer fiber.^28,29^ Moreover, the incorporation of particles into the fiber surface involves the risk that the particles may stick to the surface of the yarn and may come loose.

Therefore, a unique incorporation device was developed for capturing the added particles, which allowed for the production of composite yarns comprising a carrier yarn, a nanofiber layer, and the added particles that were fixed within a nanofiber wrapper.

## 2 Materials and Methods

### Materials

A polyester carrier yarn (PES, ELASTEX Krnov, Czech Republic) was used for the preparation of the composite yarns for technical application purposes, and Chirlac Rapid braided surgical yarn (Chirana T.Injecta, Czech Republic) was used for the preparation of composite yarns for medical applications. Polyvinyl butyral (PVB, Mowital B 60 H, Kuraray America, USA), polyamide 6 (PA 6, Ultramid B27, BASF, Germany), and polycaprolactone (PCL, Mn 80,000, Sigma-Aldrich, Germany) were used to form the nanofiber coating. The solvents used to prepare the solutions comprised ethanol, acetic acid, formic acid, sulfuric acid, and acetone (all Penta Chemicals, Czech Republic). The incorporated materials consisted of activated carbon, Sorbetin (Eco Expert, Czech Republic), and hydroxyapatite (HAP) (Sigma-Aldrich, Germany).

### Preparation

The concentrations and solvent systems for the various polymers were selected in accordance with the procedures set out in our previous publications. PVB was prepared at a concentration of 10 wt % in ethanol, and PA 6 was also prepared at a concentration of 10 wt %. Sulfuric acid was added dropwise to the spinning solution up to a total concentration of 1.8 wt % in a solvent system composed of acetic acid and formic acid at a ratio of 1:1. The final polymer, PCL, was prepared at a concentration of 10 wt % in a solvent system composed of acetic acid, formic acid, and acetone at a ratio of 1:1:1.

**Figure 1:**
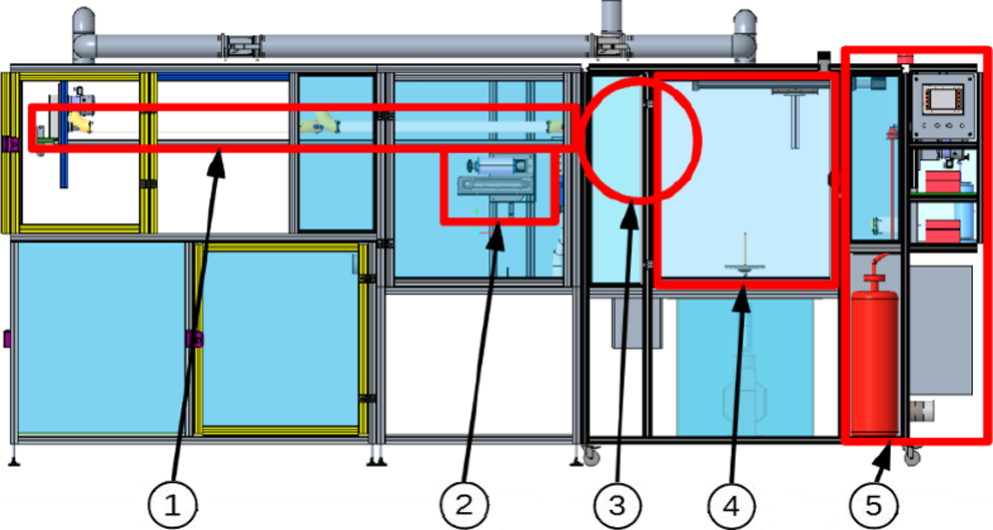
Scheme of the spinning equipment. (1) drying zone, (2) winding mechanism, (3) location of the incorporation equipment, (4) spinning zone, and (5) base yarn unwinding zone.

The resulting solutions were spun using the device shown in Figure 1; the device includes an ABB KGUG 36 high-voltage transformer and a Thalheimer-Trafowerke ESS 104 variable autotransformer. The PVB was spun at an effective voltage of 30 kV and a frequency of 50 Hz. The distance of the yarn from the spinning electrode was set at 235 mm and the yarn discharge speed at 15 m/min. The yarn was twisted using two twisting devices in order to tighten the yarn and to capture the incorporated particles. The front twisting device was set at a speed of 12 000 rpm and the rear twisting device at a working speed of 9000 rpm. The effective voltage was changed to 38 kV during the spinning of PA 6 and the speed of the discharged yarn was adjusted to 3 m/min. The bends were also changed to a front twist of 5800 rpm and a rear bend of 1500 rpm. The same settings were used for the spinning of PCL as for PA 6.

**Figure 2:**
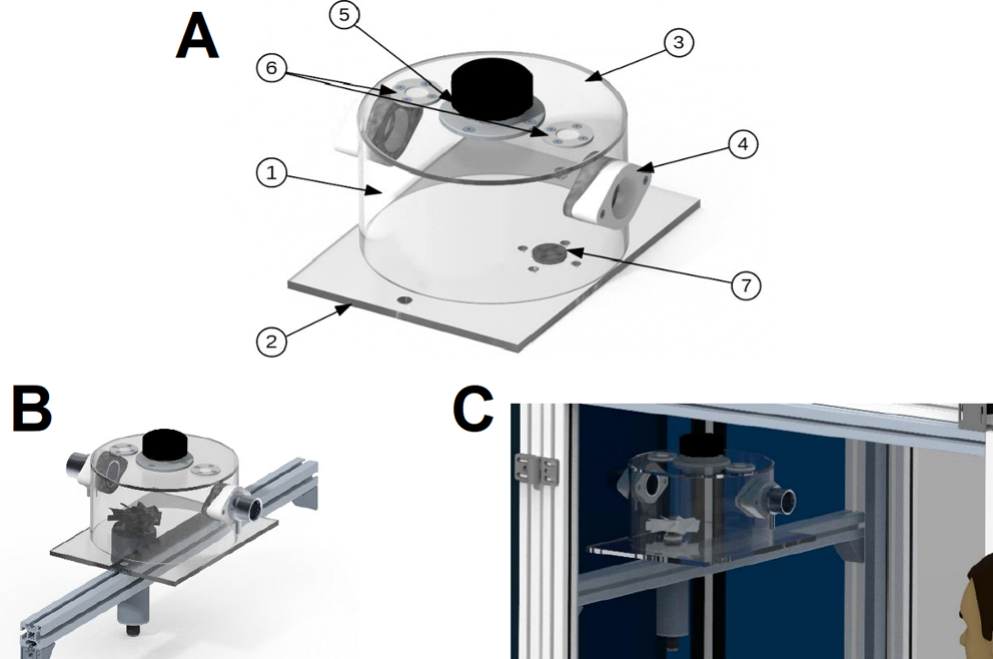
Scheme of the incorporation equipment. (A) incorporation equipment: (1) swirl chambers, (2) base, (3) lid, (4) ejector flange, (5) lid, (6) filter cloth, (7) flange; (B) incorporation chamber with the impeller; (C) incorporation chamber inside the machine.

A cylindrical chamber with an eccentrically mounted impeller, as shown in Figure 2, which acted as an aerodynamic seal, was developed for the incorporation of the small particles. The yarn was fed into the chamber through the center of the ejectors.

### Scanning Electron Microscopy and Analysis

Images of the composite yarns were taken using a scanning electron microscope (Tescan Vega 3, Czech Republic). The samples were gilded with a 10nm layer of gold using a Quorum Q150R ES device (Quorum Technologies, UK) prior to scanning. The images were taken at an accelerating voltage of 20 kV in the secondary electron detection mode. The fiber diameters were evaluated using ImageJ software (NIH, Bethesda, USA).

### Strength of the Yarns

The strength of the yarns and modified yarns were measured using a LabTest 4.050 device (Labortech instrument, Czech Republic) applying a head with a range of up to 1000 N, a loading speed of 20 mm/min, and a clamped length of 100 mm. Five samples were tested from each yarn.

## 3 Results and Discussion

### Preparation of the Composite Yarns

PES yarn and Chirlac Rapid surgical suture were used as the carrier yarns for the preparation of the composite yarns. The polymers PVB, PA 6, and PCL were used to form the nanofiber coating. The incorporated additives comprised activated carbon, HAP, and Sorbetin. Table 1 shows the distribution of the composite yarns produced according to the type of base yarn, the nanofiber sheath, the incorporated particles, and the concentration of particles in the chamber.

**Table 1:** Distribution of the Fabricated Composite Yarns

core yarn	nanofibrous shell	Incorporated particles	Concentration of particles in the swirling chamber [g/l]
PES	PVB	sorbetin	0.25
			0.5
		active carbon	0.25
			0.5
			0.75
	PA 6	sorbetin	0.25
		active carbon	0.25
			0.5
			0.75
chirlac rapid	PCL	hydroxyapatite	0.25
			0.5
			0.75

**Figure 3:**
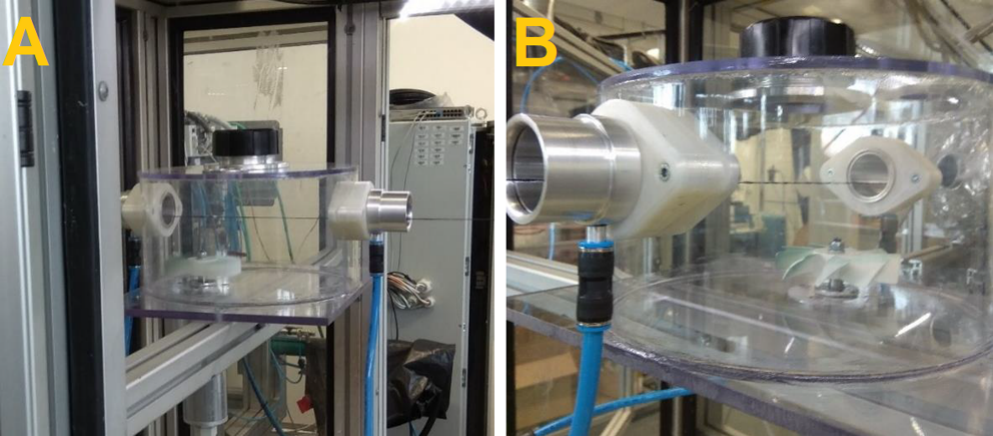
Incorporation equipment inside the machine. (A) Incorporation device located inside the machine. (B) Detail of the incorporation device with the supporting yarn.

The incorporated particles were dosed into the vortex chamber, as shown in Figure 3, where they were distributed by means of a paddle wheel. Each of the concentrations of particles in the chamber was agitated during the production process by applying three different impeller speeds, that is, 2000, 3040, and 4720 rpm.

### Morphology and Analysis

**Figure 4:**
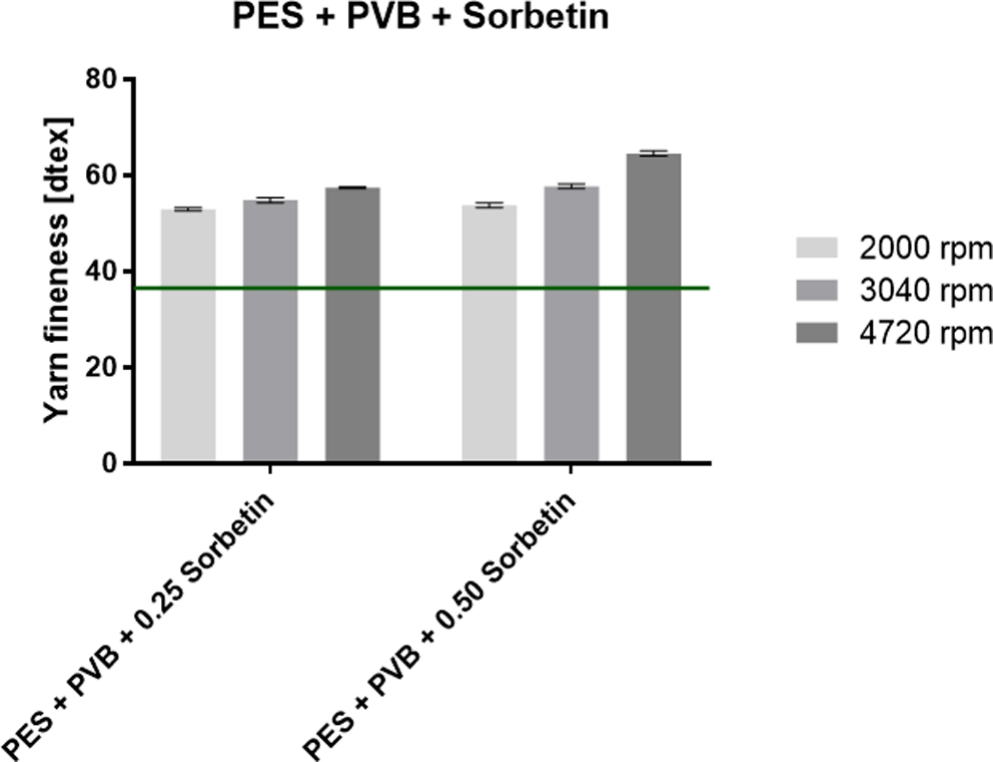
Fineness of the fabricated PES composite yarns with PVB nanofibers with the incorporated Sorbetin particles. The green line indicates the fineness of the pure PES yarn.

**Figure 5:**
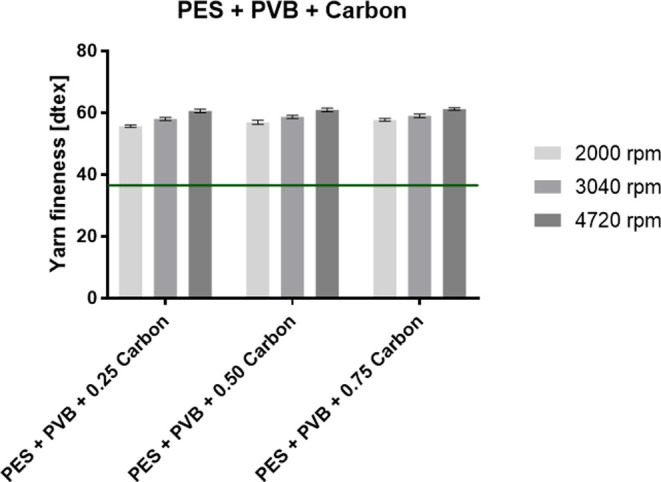
Fineness of the fabricated PES composite yarns with PVB nanofibers with the incorporated activated carbon particles. The green line indicates the fineness of the pure PES yarn.

**Figure 6:**
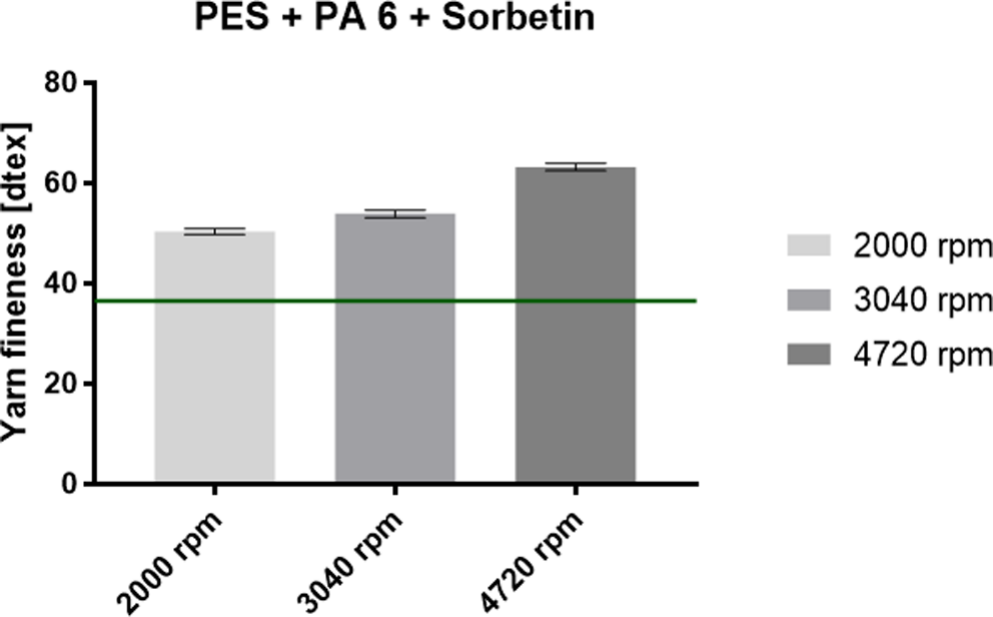
Fineness of the fabricated PES composite yarns with PA 6 nanofibers with the incorporated Sorbetin particles at a concentration of 0.25 g/l. The green line indicates the fineness of the pure PES yarn.

**Figure 7:**
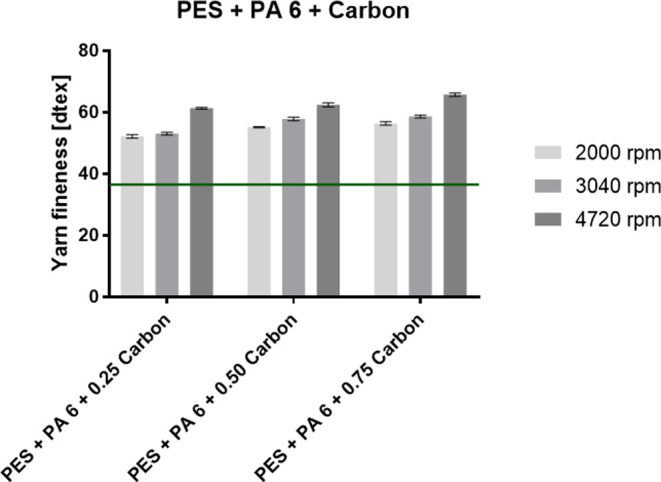
Fineness of the fabricated PES composite yarns with PA 6 nanofibers with the incorporated activated carbon particles. The green line indicates the fineness of the pure PES yarn.

The spinning of all the polymer solutions was stable, and evenly coated composite yarns were formed during the spinning process. Samples with differing concentrations of the various types of incorporated particles were prepared during the incorporation process. As previously mentioned, the concentration of incorporated particles varied in terms of both the number of particles in the incorporation chamber and the speed of the impeller. The fineness of the pure PES yarn was 36.5 dtex, whereas the PES yarn coated with PVB nanofibers evinced a fineness of 53.2 dtex. The graphs in Figures 4 and 5 show the fineness of the PES composite yarns for PVB with Sorbetin and activated carbon particles. The graphs in Figure 6 and Figure 7 show the fineness of the PES composite yarns for PA 6 with Sorbetin and activated carbon particles.

**Figure 8:**
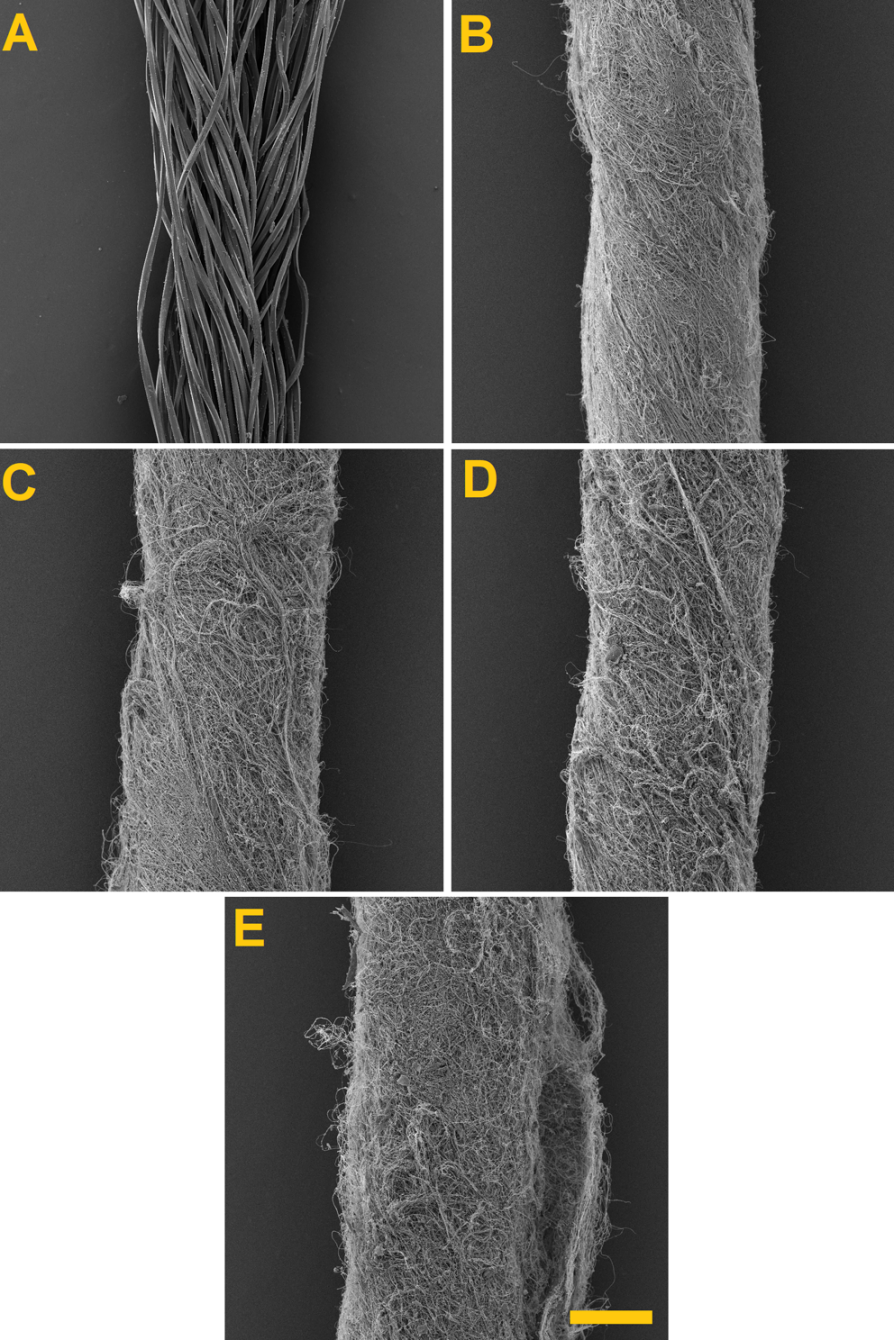
SEM images of the yarn and fabricated composite yarns. (A) Yarn PES, (B) yarn PES + PVB, (C) yarn PES + PVB + 0.75 carbon, 2000 rpm, (D) yarn PES + PVB + 0.75 carbon, 3040 rpm, and (E) yarn PES + PVB + 0.75 carbon, 4720 rpm. Scale 200 μm.

The examination of the fabricated composite yarns revealed that the particles were present across the whole of the cross-sections of the nanofiber sheaths of the yarns; that is, the supplied particles occurred both on the surface of the yarn and within the whole of the nanofiber wrapper. Figure 8 shows the SEM images used for the comparison of the fabricated composite yarns.

**Figure 9:**
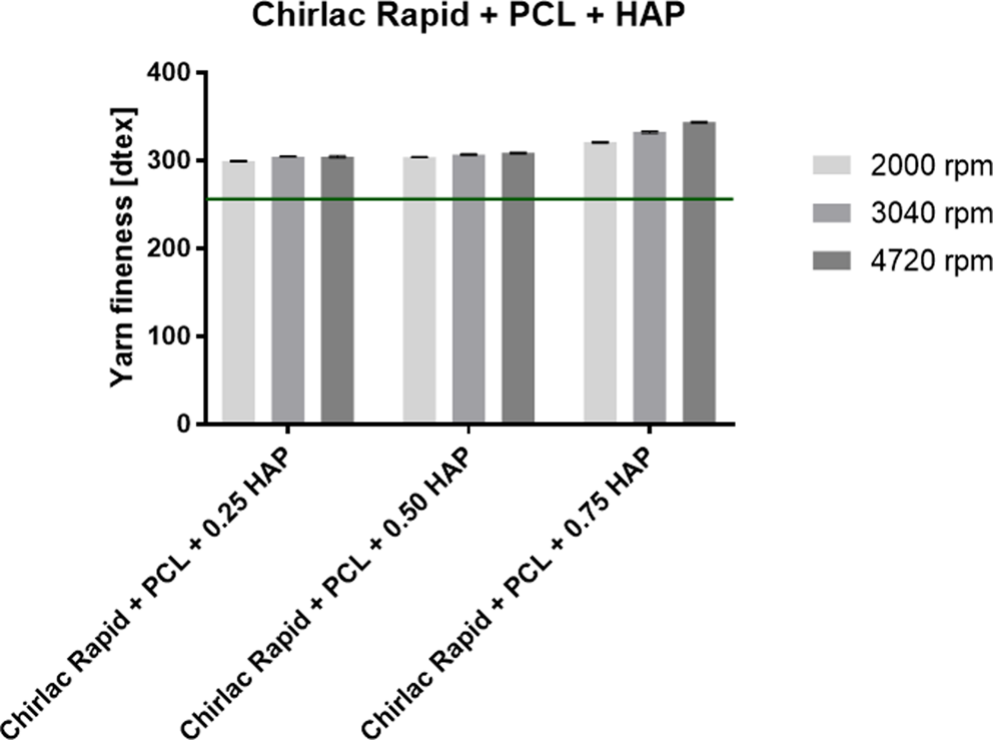
Fineness of the fabricated composite surgical yarns with PCL nanofibers with the incorporated HAP particles. The green line indicates the fineness of the pure Chirlac Rapid surgical yarn.

**Figure 10:**
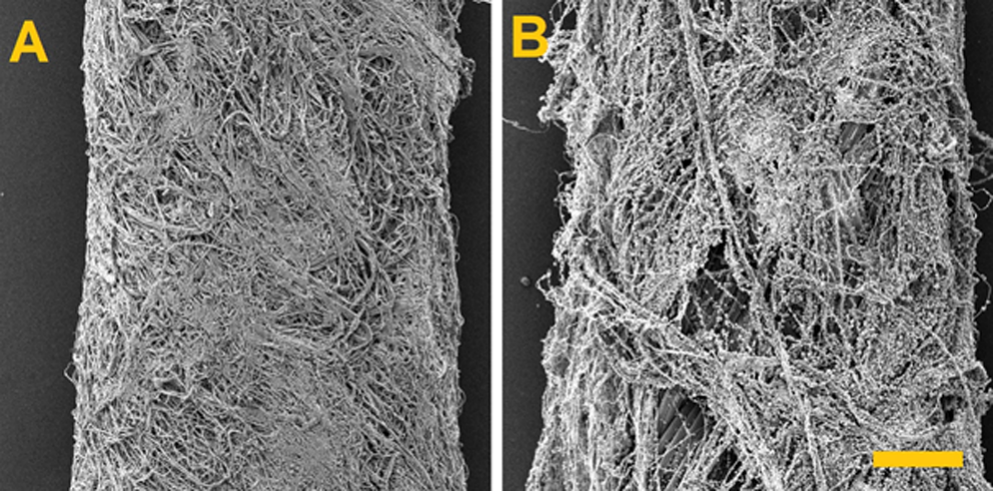
SEM images of the surgical yarns. (A) Without incorporated particles and (B) with incorporated HAP particles.Scale 200 μm.

We subsequently initiated the production of composite surgical yarns aimed at determining whether yarns destined for medical applications could be produced via this approach. PCL was selected for spinning and HAP for incorporation. The graph in Figure 9 provides a comparison of the fineness of the composite surgical yarns produced. Figure 10 provides a comparison of a surgical yarn without incorporated particles and with incorporated HAP particles.

It was found during the production of the composite yarns that the particle size affects the incorporation process. The Sorbetin particles used in the study had a particle size of 308.8 ± 87.5 μm. During the incorporation of these spatially significant particles, the uneven flow of particles was observed inside the chamber, which resulted in the uneven incorporation of particles on the composite yarn. However, the flow of activated carbon (particle size 1.628 ± 0.632 μm) and HAP (particle size 1.838 ± 0.708 μm) particles in the chamber was observed to be even, which resulted in the even incorporation of the particles on the composite yarn.

### Strength of the Yarns

**Figure 11:**
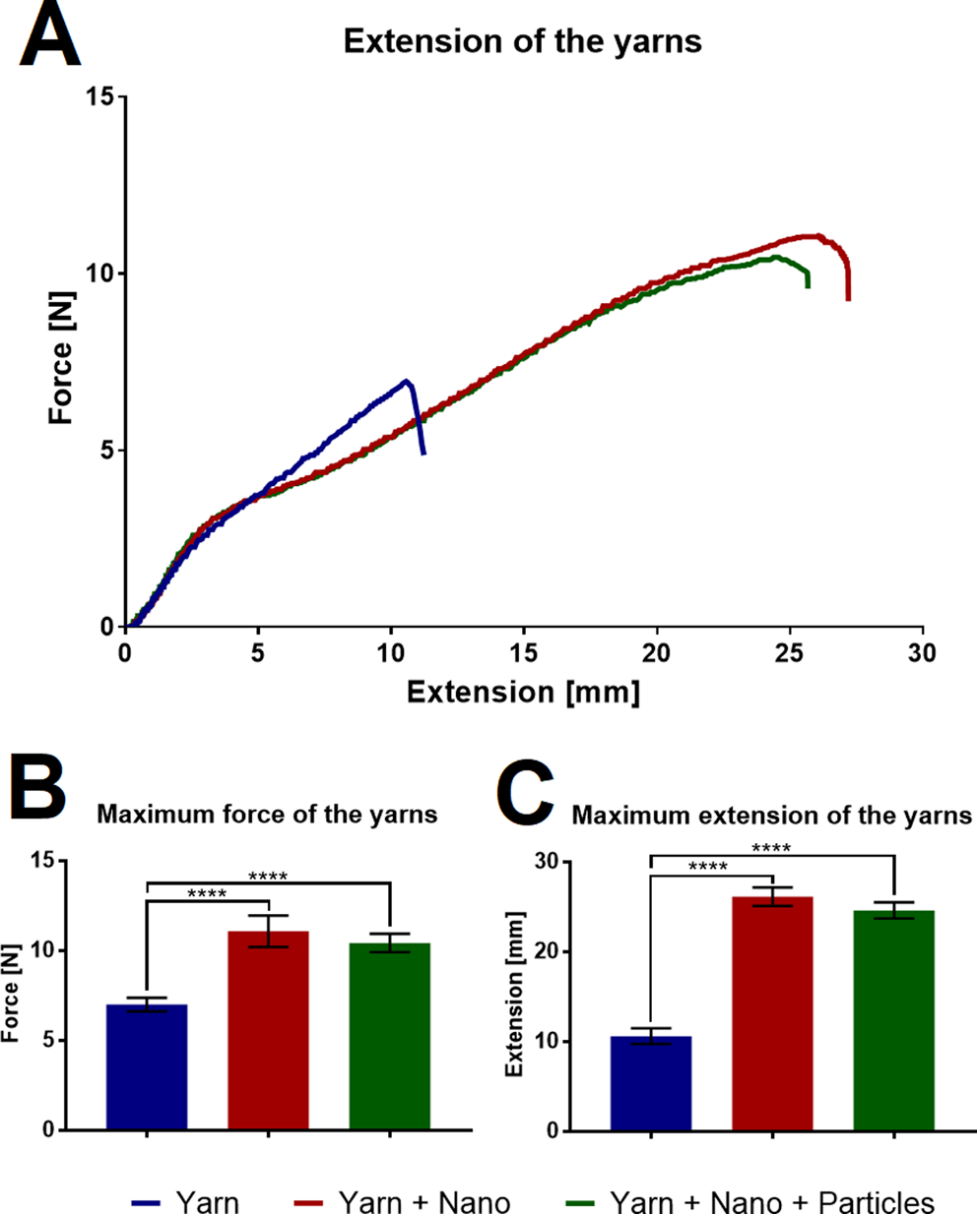
Mechanical testing of the yarns. (A) Results of the tensile testing of the yarn and modified yarns. (B) Maximum strength of the yarns. (C) Maximum elongation of the yarns. 95% confidence interval (CI); ****p < 0.0001.

To determine whether the nanofiber coating and added particles affect the mechanical properties of the yarns, the strength of the used PES yarn, PES yarn + PVB nanofiber sheet, and PES yarn + PVB nanofiber sheet + 0.75 carbon was measured. The graph in Figure 11 shows the tensile curves and the comparison of the maximum strength and maximum elongation that the yarns are able to achieve before breaking.

According to the results, it is evident that the nanofiber coating significantly increases the strength and ductility of the yarn. Activated carbon particles lead to a slight decrease in yarn strength and ductility compared to yarn with only nanofiber coating, but it is still significantly higher than pure yarn. The results indicate that composite yarns will be easier to handle in subsequent applications.

### Preparation of the Candle Filter

Composite nanofiber yarns spun onto so-called candle filters are commonly used for filtration purposes. The advantage of candle filters lies in the fact that it is technologically relatively simple to wind a spatial structure with the required thickness and with defined properties from yarn through the thickness of the wound layer. Thus, it is possible to ensure that filtration takes place not only on the surface of the filter but through the entire volume of the filter layer.

Both the barrier principle caused by the small distances between adjacent fibers and, above all, the effect of a large surface area of nanofibers and particles apply when employing nanofibers for filtering purposes. It is possible to use the precise cross-winding or the digital winding (consisting of several differing precise cross-winding layers) approaches. Closed winding is applied that acts to form a typical diamond pattern. The most important parameter comprises the distance between the adjacent wraps, which should be chosen in accordance with the diameter of the yarn and the required filtered fluid flow rate.^30^

**Figure 12:**
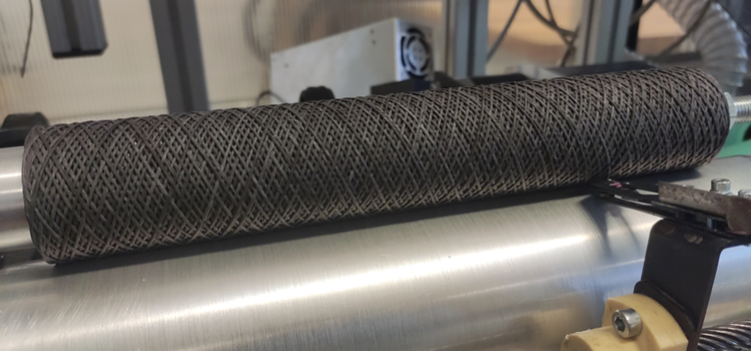
Composite candle filter comprising a carrier PES yarn, PVB nanofibers, and activated carbon particles.

**Figure 13:**
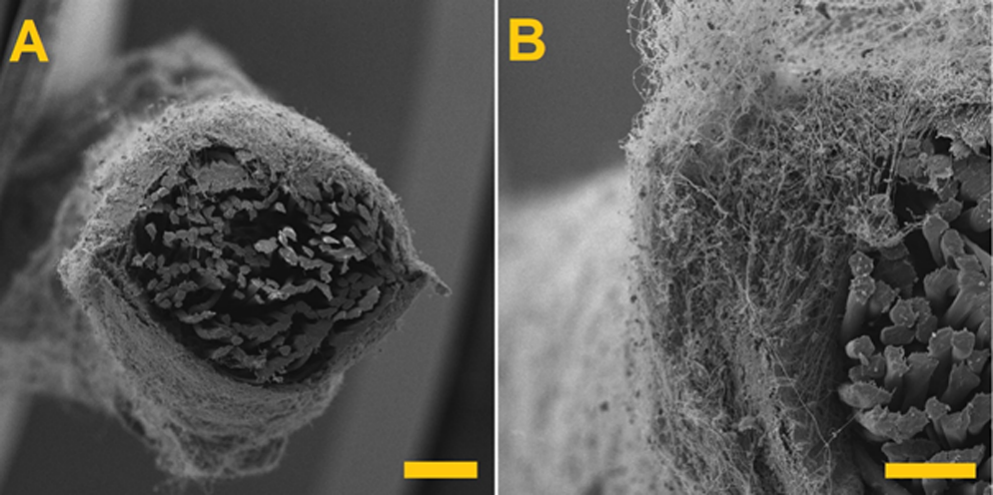
Cross-section of a composite nanofiber yarn. (A) Cross-section of whole composite yarn. Scale 200 μm. (B) Detail of cross-section of composite yarn. Scale 100 μm.

A specially designed winding device was used for the winding of the filters, which allowed for the precise electronic control of the winding process using step-by-step motors. It was thus possible to create the desired winding structure, while the twisting ratio and the associated distance between the adjacent wraps could be changed according to the requirements of the filter parameters. In addition, the device allows for the definition of the hardness of the coil via both the regulation of the tensile force during winding and the electronic regulation of the pressing force between the spool and the support roller. Figure 12 shows a manufactured candle filter comprising a carrier PES yarn, PVB nanofibers, and activated carbon particles. The candle filter was manufactured at a particle concentration of 0.75 g/l in the incorporation chamber and a paddle speed of 4720 rpm. Figure 13 shows a detail of a cross-section of a composite nanofiber yarn.

The candle filter was fabricated by winding the composite yarn onto a perforated spool with dimensions of 250 mm in length and 28 mm in diameter. 1500 m of the composite yarn was used to produce the candle filter. The wall thickness of the candle filter with the composite yarn was 16 mm.

## 4 Conclusions

Modified composite yarns, the production, and processing of which have been described in several of our previous publications were considered in the study. The research involved the modification of composite yarns using fine particles aimed at significantly enhancing their utility value, for example, the enhancement of the filtration efficiency of filters using composite yarns with incorporated particles and the use of such yarns in medical applications. The study included the modification of composite yarns using incorporated Sorbetin, activated carbon, and HAP particles. Sorbetin particles with a size of 308.8 ± 87.5 μm were found to be too large for use in the impeller chamber; the flow of Sorbetin particles was observed to be uneven in the chamber, which resulted in the uneven incorporation of the particles. However, the activated carbon and HAP particles with sizes of 1.628 ± 0.632 am and 1.838 ± 0.708 μm, respectively, were observed to be evenly distributed in the incorporation chamber, which resulted in the uniform incorporation of the particles on the composite yarn. The fineness of the modified composite yarns is influenced by both the concentration of particles in the incorporation chamber and the rotation speed of the impeller in the chamber. The study involved the testing of three different particle concentrations in the incorporation chamber, that is, 0.25, 0.5, and 0.75 g/l. The application of higher concentrations of particles in the incorporation chamber led to increases in the fineness of the resulting modified composite yarns. Impeller speeds of 2000, 3040, and 4720 rpm were applied. As with the higher concentration of particles, the application of a higher impeller rotational speed acted to enhance the fineness of the resulting modified composite yarns. The final part of the study focused on the production of a modified composite candle filter comprising a carrier PES yarn, PVB nanofibers, and activated carbon particles. It is recommended that future research should focus on the testing and application of candle filters produced via this approach.
